# Successful pregnancy and term delivery after treatment of unicornuate uterus with non-communicating rudimentary horn pregnancy with local methotrexate injection followed by laparoscopic resection: a case report and literature review

**DOI:** 10.1186/s12884-021-04195-5

**Published:** 2021-10-26

**Authors:** Makiko Ueda, Kuniaki Ota, Toshifumi Takahashi, Satoshi Suzuki, Daisuke Suzuki, Hyo Kyozuka, Masatoshi Jimbo, Shu Soeda, Takafumi Watanabe, Keiya Fujimori

**Affiliations:** 1 Department of Obstetrics and Gynecology, Fukushima medical University, Fukushima, 960-1295 Japan; 2 Fukushima Medical Center for Children and Women, Fukushima medical University, 1 Hikarigaoka, Fukushima City, Fukushima, 960-1295 Japan; 3 Department of Obstetrics and Gynecology, Toho University, Tokyo, 143-8541 Japan

**Keywords:** Rudimentary horn, Ectopic pregnancy, Methotrexate, Laparoscopy, Case report

## Abstract

**Background:**

Pregnancy in a rudimentary horn is an extremely rare type of ectopic pregnancy. A rudimentary uterine horn pregnancy is associated with a risk of spontaneous rupture and bleeding during surgery due to the increased uterine blood flow. Recent advances in imaging modalities have enabled laparoscopic surgery to be performed in cases without rupture in the early stages of pregnancy. However, there are few reports of successful pregnancies and deliveries after treatment of rudimentary horn pregnancies. We report the successful management of a case of non-communicating rudimentary horn pregnancy by local injection of methotrexate followed by complete laparoscopic excision along with a review of the literature.

**Case presentation:**

The patient was a 29-year-old Japanese woman, gravida 2, nullipara. She was diagnosed with a left unicornuate uterus with a right non-communicating rudimentary horn on hysterosalpingography and magnetic resonance imaging. A gestational sac with a heartbeat was observed in the right rudimentary uterine horn at 6 weeks of gestation. A diagnosis of ectopic pregnancy in a non-communicating rudimentary horn was made. Color Doppler detected multiple blood flow signals around the gestational sac, which were clearly increased compared to the left unicornuate uterus. Her serum human chorionic gonadotropin level was 104,619 mIU/ml. A 100 mg methotrexate injection into the gestational sac was administered, and laparoscopic surgery was performed on day 48 after the methotrexate treatment. The right rudimentary horn and fallopian tube were successfully excised with minimal bleeding. A spontaneous normal pregnancy was established 6 months after the surgery. The pregnancy was uneventful, and a baby girl was born by elective cesarean section at 38w0d.

**Conclusion:**

Combined local methotrexate injection and laparoscopic surgery are safe treatment options for patients with a unicornuate uterus with a non-communicating rudimentary horn pregnancy.

## Background

A unicornuate uterus with a rudimentary horn arises from the arrested development of one of the Mullerian ducts, and is found in 0.4% women [1]. There are two types of unicornuate uteruses with communicating and non-communicating rudimentary horns [2]. If a rudimentary horn has a cavity with endometrium, it may serve as an implantation site for pregnancy [3, 4]. Pregnancy in a rudimentary horn is an extremely rare type of ectopic pregnancy, ranging from 1/100,000 to 1/140,000 pregnancies [4].

Regarding the natural history of a pregnancy in the rudimentary horn, it is often diagnosed during laparotomy when the gestational horn ruptures in the first or second trimester of pregnancy, causing severe, life-threatening hemoperitoneum [5, 6]. Recent advances in diagnostic imaging modalities, such as ultrasound and magnetic resource imaging (MRI), have made it possible to diagnose these pregnancies before occurrence of rupture [7, 8]. Early diagnosis can help in performing laparoscopic surgical treatment before rupture [7, 9].

However, when a rudimentary horn is surgically excised, there is a concern about the risk of bleeding due to increased blood flow to the uterus due to pregnancy. There are several reports on medical management using systemic or local methotrexate (MTX) to reduce the uterine blood flow due to pregnancy, followed by surgical resection of the rudimentary horn pregnancy [10–16]. Nevertheless, there are few reports of pregnancies and deliveries after treatment of a rudimentary horn pregnancy [17].

Here, we report a case who had a successful pregnancy and term delivery after local injection of MTX, followed by laparoscopic excision of a non-communicating rudimentary horn pregnancy along with a literature review.

## Case presentation

**Fig. 1:**
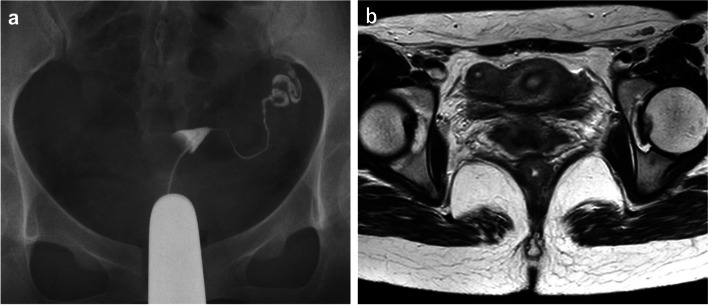
Diagnosis of unicornuate uterus with a non-communicating rudimentary horn. **a** Image of hysterosalpingography. **b** T2-weighted transverse magnetic resonance imaging

**Fig. 2:**
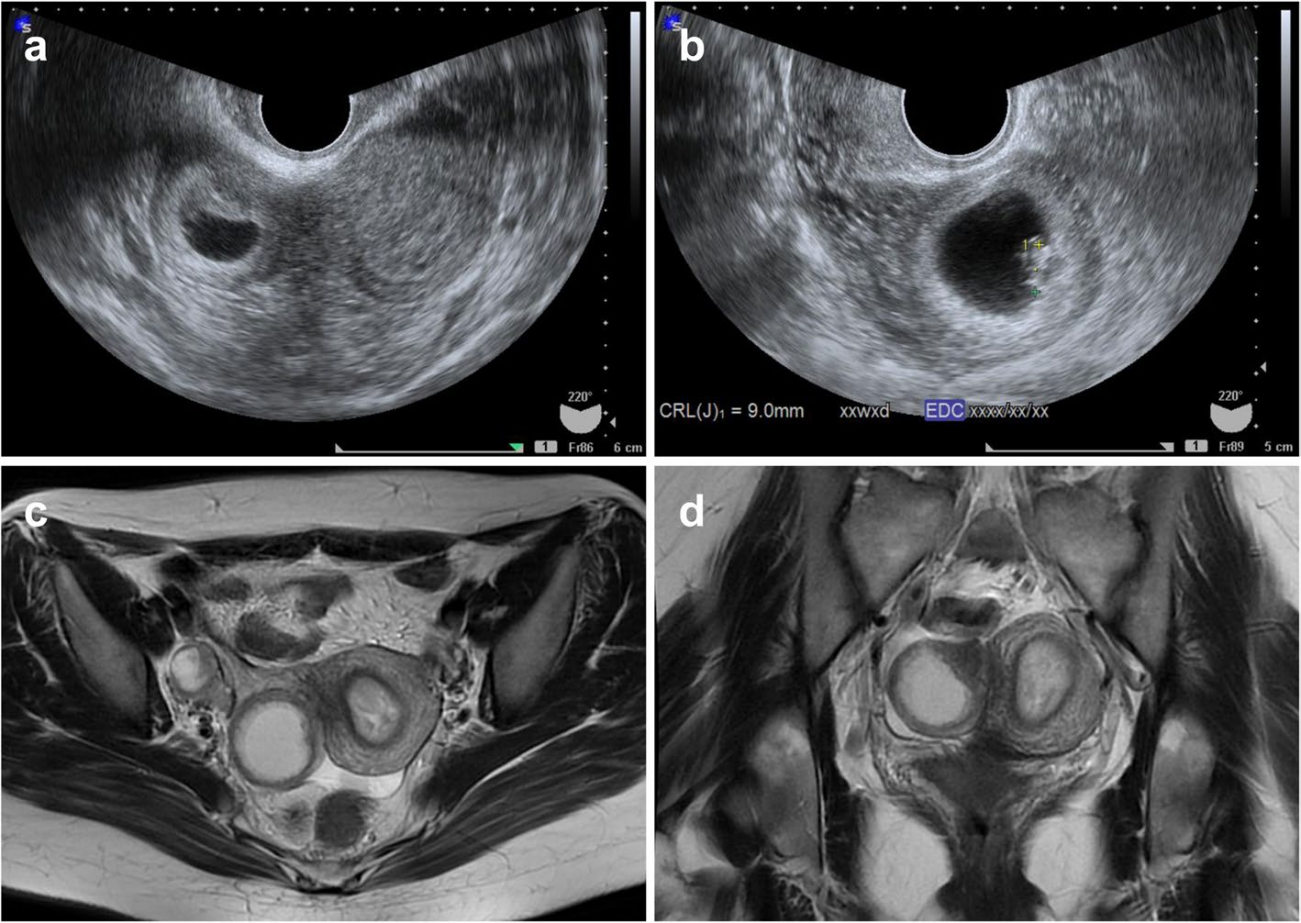
Images showing a left unicornuate uterus with a right noncommunicating rudimentary horn pregnancy. On transvaginal ultrasonography, **a** a gestational sac was observed in the right rudimentary horn, and thickened endometrium was observed in the left unicornuate uterus at 6 weeks of gestation; **b** a fetus of 9.0 mm size with a heartbeat was observed at 7 weeks of gestation. **c** T2-weighted transverse magnetic resonance imaging at 7 weeks of gestation **d** T2-weighted coronal magnetic resonance imaging at 7 weeks of gestation

The patient was a 29-year-old Japanese woman, gravida 2, nullipara. She had no significant medical history other than two spontaneous miscarriages. She had been diagnosed with a unicornuate uterus with a non-communicating rudimentary horn by hysterosalpingography (HSG) and MRI after a miscarriage at 28 years of age (Fig. 1). She presented to the obstetrics and gynecology clinic with the chief complaint of a 10-day delay in expected menstruation, along with a positive urine pregnancy test. She was considered to be at 5 weeks and 3 days of gestation according to her last menstrual period. Transvaginal ultrasonography showed no gestational sac in the unicornuate uterus, but an 18 mm-gestational sac was observed in what appeared to be a non-communicating rudimentary horn. At 6 weeks and 2 days of gestation, the patient was referred to our hospital with a diagnosis of ectopic pregnancy. She had no symptoms of genital bleeding or abdominal pain. On visual examination using a vaginal speculum, there was a single cervix with erosion and a moderate amount of white discharge. Bimanual internal examination was not performed because of the risk of rupture of the ectopic pregnancy. Transvaginal ultrasonography revealed no gestational sac in the left unicornuate uterus, and a 21 mm-gestational sac with a positive fetal heartbeat in the right non-communicating rudimentary horn (Fig. 2a). Consequently, a diagnosis of ectopic pregnancy in a non-communicating rudimentary horn was made. The patient and her husband were informed that it would be difficult to continue the pregnancy and that surgical removal of the pregnancy site was necessary. The patient was admitted for treatment at 7 weeks and 1 day of gestation. Transvaginal ultrasonography showed a fetal heartbeat positive 9 mm fetus in a 27 mm-gestational sac in the right rudimentary horn (Fig. 2b), and color Doppler detected multiple blood flow signals around the gestational sac, which were clearly increased compared to the left unicornuate uterus. MRI revealed evidence of right rudimentary horn pregnancy consistent with the transvaginal ultrasonography findings; the right rudimentary horn was enlarged, and its cavity demonstrated a high signal in T2-weighted image, which was thought to correspond to the gestational sac. The corpus luteum cyst was in the right ovary, and the left unicornuate uterus showed a thickened endometrium (Fig. 2c and d). The serum level of human chorionic gonadotropin (hCG) was 104,619 mIU/ml. Considering the abundant blood flow around the pregnancy site and the risk of bleeding during surgery, we planned to perform surgery after performing a therapeutic abortion with local injection of MTX into the pregnancy site.

The patient was admitted to the hospital at 7 weeks and 2 days of gestation, and received a local MTX injection at the site of pregnancy. Under transvaginal ultrasound guidance, a 20-gauge needle (oocyte aspiration needle, OPU-NP20G325B, KITAZATO CORPORATION, Tokyo, Japan) was used to puncture the gestational sac in the right rudimentary horn, and 8 ml of fluid was aspirated. The fetus was then punctured to confirm that the fetal heartbeat was negative. Subsequently, 100 mg of MTX dissolved in 4 ml saline was injected topically into the chorionic area, the needle was removed, and color Doppler was used to confirm the blood flow signal from the puncture site. Since bleeding from the uterine puncture site into the abdominal cavity was suspected, the puncture site was compressed with an ultrasound probe to stop bleeding. The time taken for these procedures was 10 min.

**Fig. 3:**
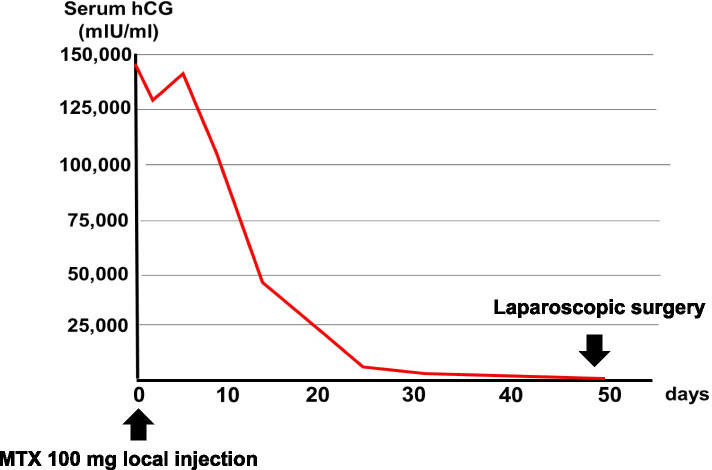
Changes in the serum human chorionic gonadotropin (hCG) level after the methotrexate local injection

After MTX treatment, the pregnancy site was closely observed by transvaginal ultrasound, and serum hCG levels were closely measured. A transient hCG elevation was observed after MTX treatment, but the hCG level subsequently decreased; on postoperative day 30, the hCG level dropped to 1587 mIU/ml (Fig. 3). Color Doppler showed that the blood flow signals from around the gestational sac in the right rudimentary horn were clearly decreased compared to those before the treatment.

**Fig. 4:**
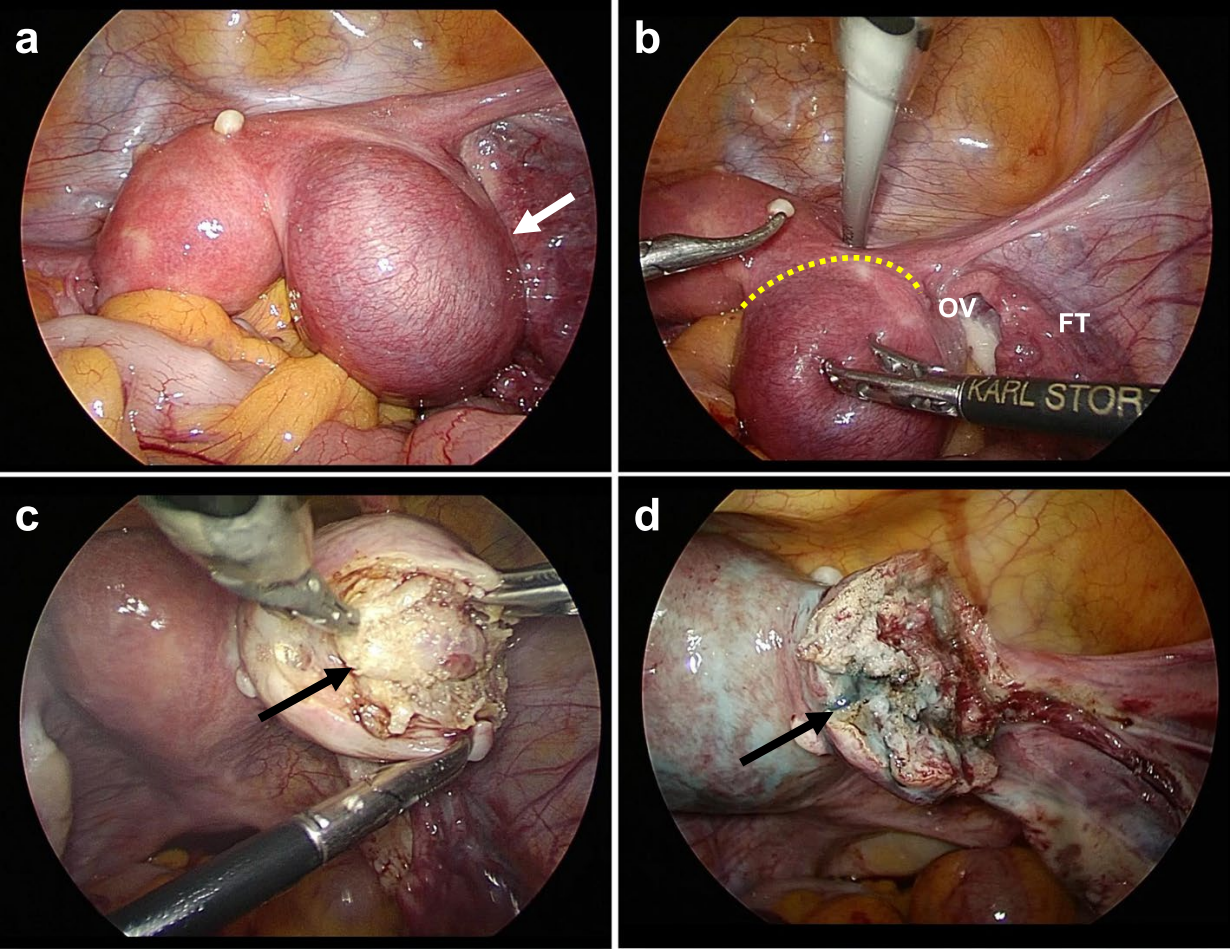
Photographs of laparoscopic surgery. **a** Laparoscopic findings of right pregnant rudimentary horn and left unicornuate uterus. The white arrow indicates the pregnancy in the right rudimentary horn. **b** The yellow dotted line shows the attachment between the left uterus and right rudimentary horn. **c** The black arrow indicates the enucleation of the gestational sac after incision of the uterine muscle. **d** Photograph of the resected stump of the rudimentary horn. The black arrow indicates spillage of the indigo carmine solution injected into the left uterus

Laparoscopic resection of the right rudimentary horn 48 days after the MTX treatment was performed. We created a 12-mm trocar port in the umbilicus for 10 mm straight optics, and three 5-mm trocar ports in the lower abdomen for laparoscopic trocar port placement. Observation of the abdominal cavity revealed no ascites, bleeding, or adhesions. The right pregnant rudimentary horn was enlarged and more than twice the size of the left unicornuate uterus (Fig. 4a). The attachment between the right rudimentary horn and the left unicornuate uterus was several centimeters in size (Fig. 4b). A 21-gauge needle was inserted through the abdominal wall into the site of the right rudimentary horn directly, and diluted Pitressin (0.1 U/ml, 20 units diluted in 200 mL saline) was injected to decrease the bleeding. An ultrasonic scalpel (Harmonic Ace, Ethicon, Tokyo, Japan) was placed inside the pregnant right uterine wall, exposing and enucleating the gestational sac portion, with minimal bleeding (Fig. 4c). The rudimentary horn and right fallopian tube were resected from the site of attachment to the left uterus. After resecting the right rudimentary horn, a balloon catheter was placed in the left uterine lumen. An indigo carmine solution was injected to confirm communication with the right rudimentary horn. Although leakage of the indigo carmine solution was observed into the muscular layer of the resected uterus, when we attempted to insert a sonde through the indigo carmine seepage site, we could not find an obvious communicating channel to the left uterus (Fig. 4d). The resection site was Z-sutured using biodegradable poly-p-dioxanone (PDS-II, Ethicon, Johnson and Johnson, Tokyo, Japan). A peritoneal endometriosis lesion was electrocauterized, and a submucosal myoma was enucleated. Finally, the surgery was completed with the application of a regenerated cellulose adhesion barrier (Interceed, Ethicon, Johnson and Johnson, Tokyo, Japan) to cover the right rudimentary horn resection site. The operative time was 123 min, and the amount of blood loss was 15 ml. Histopathological findings revealed degenerated chorionic and decidual tissues in the excised gestational sac.

No postoperative complications were observed, and the patient was discharged on the 4th postoperative day. The serum hCG levels decreased steadily; on postoperative day 17, the hCG level was 3.3 mIU/ml. Menstruation resumed 26 days after the operation, and a spontaneous normal pregnancy occurred 6 months after the surgery. The gestational course was uneventful, and a baby girl was born by elective cesarean section at 38w0d, weighing 2546 g with an APGAR score of 8 at 1 min. During surgery, no thinning of the myometrium or adhesions at the site of resection of the right rudimentary horn were observed.

## Discussion and conclusions

The mechanism of pregnancy in a non-communicating rudimentary horn with a unicornuate uterus may be the intraperitoneal migration of spermatozoa or fertilized oocytes [18]. In other words, spermatozoa ejaculated vaginally migrate into the abdominal cavity through the unicornuate uterus, and may be then fertilized in the fallopian tube on the side of the rudimentary horn, followed by implantation. Another possibility is that the oocytes ovulated from either the left or right ovary were fertilized by spermatozoa that passed through the unicornuate uterus, and the fertilized oocytes migrated through the abdominal cavity followed by implantation in the rudimentary horn.

In this case, since the corpus luteum was ipsilateral to the right rudimentary horn, it was assumed that the spermatozoa migrated through the left unicornuate uterus into the abdominal cavity and were then fertilized the oocyte in the right fallopian tube. After laparoscopic resection of the pregnant rudimentary horn, the indigo carmine dye flowed out from the unicornuate uterus at the resection site (Fig. 4). Therefore, the possibility of communication between the left unicornuate uterus and right rudimentary horn was considered. However, the possibility that the myometrium on the side of the unicornuate uterus was damaged during excision of the rudimentary horn could not be ruled out.

Early diagnosis of rudimentary horn pregnancy is important because rupture can cause intraperitoneal hemorrhage, which can be fatal for the patient. The decrease in the mortality rate from 23 to 0.5% [19] in recent years may be due to the advances in imaging modalities such as ultrasound and MRI performed before the pregnancy [20]. To diagnose a rudimentary horn pregnancy, it is necessary to differentiate it from a bicornuate pregnancy when the patient is examined for the first time. Before pregnancy, transvaginal ultrasound and MRI can be useful in distinguishing a bicornuate uterus from a unicornuate uterus with a rudimentary horn because the size of the rudimentary horn is smaller than that of a normal uterus. However, for diagnosis during a pregnancy, some researchers have claimed that the preoperative diagnosis of a rudimentary horn pregnancy was achieved in only 5% of all reported cases [21] and the sensitivity of ultrasound was 26% preoperatively [22]. Blancafort et al. reported a patient with a unicornuate uterus with a pregnancy in the non-communicating rudimentary horn who underwent preoperative 3-dimensional (3-D) transvaginal ultrasound followed by laparoscopic resection of the pregnant-rudimentary horn [23]. The usefulness of 3-D ultrasonography for the diagnosis of uterine malformations has been reported by the European Society of Human Reproduction and Embryology-European Society for Gynaecological Endoscopy [24].

However, after 7 weeks of gestation, when the fetus is detected by transvaginal ultrasound, it is more difficult to differentiate between a rudimentary horn and a bicornuate uterus because the examiner is more concerned about the development of the fetus. The presence of dysmenorrhea in the menstrual history and a unilateral endometrial cyst can trigger the suspicion of the existence of a non-communicating rudimentary horn [25]. The possibility of a rudimentary horn pregnancy should always be considered in pregnancies with two uterine cavities.

Upon diagnosis of a pregnancy in a rudimentary horn, the standard treatment involves immediate excision of the pregnant rudimentary horn for avoiding recurrence of pregnancy, removing the cause of dysmenorrhea, and preventing possible endometriosis [4]. In recent years, laparoscopic surgery has been the standard treatment for rudimentary horn pregnancy in hemodynamically stable patients [7, 9]. During resection of a pregnant-rudimentary horn, bleeding is a major surgical risk because the pregnant uterus has abundant blood flow. To reduce the risk of bleeding during surgery, medical treatments, such as MTX injection, either locally or systemically, to reduce the blood flow to the pregnant uterus followed by surgical resection, have been attempted. Edelman et al. reported the first case of preoperative treatment with a single dose of systemic MTX injection, followed by successful laparoscopic resection of the pregnant rudimentary horn [10].

A summary of the combined medical and surgical management of a rudimentary horn pregnancy is shown in Table 1. Nine cases, including ours, have been reported [10–16]. Four of the nine cases (44%) had already been diagnosed as having a unicornuate uterus with a non-communicating rudimentary horn before the rudimentary horn pregnancy. In all cases, the diagnosis of rudimentary horn pregnancy was confirmed before treatment in the early stage of pregnancy (median diagnosis at 8 weeks of gestation) before occurrence of rupture. The medical treatment consisted of MTX only in four out of nine cases, while MTX was given along with a therapeutic abortion with KCL or lidocaine injection given locally to the fetus in five cases. The waiting period from medical treatment to surgery ranged from 2 days to 6 months, and all nine patients underwent laparoscopic excision of the pregnant-rudimentary horn. In three of the four cases where the amount of bleeding was described, the bleeding was small. All patients who underwent laparoscopic surgery were treated safely without any operative complications.

**Table 1:** Cases of combined medical and surgical treatment of a unicornuate uterus with a non-communicating rudimentary horn pregnancy

Authors (years)	Age (years)	GW (weeks)	Imaging modality for diagnosis of RH	Preoperative diagnosis	Rupture	hCG levels at diagnosis or treatment (mIU/ml)	Medical treatment	Surgery	Interval between medical treatment and surgery	Connection between RH and unicornuate uterus	Operative bleeding/complications
Edelman et al. (2003) [10]	24	7	US	Right RHP	No	N.A.	MTX IM, misoprostol	LS-RHE	6 months	N.A.	N.A./none
Cutner et al. (2004) [11]	32	N.A.	Laparoscopy^a^	Right RHP	No	N.A.	Intracardiac KCl inj., MTX IM, goserelin	LS-RHE	2 months	Fibrous	NA/none
Cutner et al. (2004) [11]	32	12	3D-US	Right RHP	No	71,580	Intracardiac KCl inj., MTX IM, lueprolelin	LS-RHE + salpingectomy	6 months	N.A.	NA/none
Park et al. (2007) [12]	36	8	HSG^a^, US	Right RHP	No	89,000	Intracardiac KCl inj., MTX local inj.	LS-RHE + salpingectomy	6 weeks	Band (1.5 cm)	< 50 ml/none
Suzuki et al. (2011) [13]	19	8	US, MRI, 3D-CT	Left RHP (twin)	No	40,199	MTX local inj. and IM	LS-RHE + saplingectomy	109 days	Broad attachment	140 ml/ none
Lennox et al. (2013) [14]	28	16	US, MRI	Left RHP	No	N.A.	Intracardiac KCl inj.	LS-RHE + salpingectomy	2 days	Fibrous band (3-4 cm)	Minimal/none
Herchelroath et al. (2018) [15]	31	7	US^a^	Right RHP	No	123,523	Intracardiac lidocaine inj., MTX local inj.	LS-RHE + salpingectomy	1 month	Band	N.A./none
Rodirigues et al. (2019) [16]	34	6	3D-US, MRI	Right RHP	No	58,536	MTX local inj.	LS-RHE + salpingectomy	3 months	Fibrous band	N.A./none
Present case	30	6	HSG^a^, MRI^a^	Right RHP	No	104,619	MTX local inj.	LS-RHE + salpingectomy	48 days	Broad attachment	< 50 ml/none

*Abbreviations*: *GW* gestational weeks, *hCG* human chorionic gonadotropin, *RH* right horn, *US* ultrasonography, *3D-US* 3-dimensional ultrasonography, *HSG* hysterosalpingography, *MRI* magnetic resource imaging, *3D-CT* 3-dimensional computed tomography, *RHP* rudimentary horn pregnancy, *MTX* methotrexate, *LS-RHE* laparoscopic rudimentary horn excision, *NA* not applicable

^a^Imaging studies conducted before pregnancy

In conclusion, combined local methotrexate injection and laparoscopic surgery are safe treatment options for patients with a unicornuate uterus with a non-communicating rudimentary horn pregnancy. It is important to be aware of the possibility of a rudimentary horn pregnancy in cases with a unilateral uterine pregnancy in presence of two uterine cavities, and a detailed menstrual history should be obtained along with careful imaging in the early stages of pregnancy.

## Data Availability

Not applicable.

## Abbreviations

hCG: Human chorionic gonadotropin
HSG: Hysterosalpingography
MTX: Methotrexate
MRI: Magnetic resource imaging
3-D: 3-dimensional
